# Supplementary material for: Reversible Disulfide Bond Cross-Links as Tunable Levers of Phase Separation in Designer Biomolecular Condensates
Source: J Am Chem Soc. 2024 Aug 28;146(36):25299–311. doi: 10.1021/jacs.4c09557 (PMC11403603; doi:10.1021/jacs.4c09557)
Supplement: Supplementary file 1 — ja4c09557_si_001.pdf [file ja4c09557_si_001.pdf]

Reversible disulfide bond crosslinks as tunable levers of phase separation in designer biomolecular condensates.

Malay Mondal[1,4], Penelope E. Jankoski[2], Landon D. Lee[1], Daniel M. Dinakarapandian[3], Tzu-Ying Chiu[3], Windfield S. Swetman[2], Hongwei Wu[3], Anant K. Paravastu[3], Tristan D. Clemons*[,2,4], and Vijayaraghavan Rangachari*[,1,4].

SUPPLEMENTARY INFORMATION

SUPPLEMENTARY FIGURES

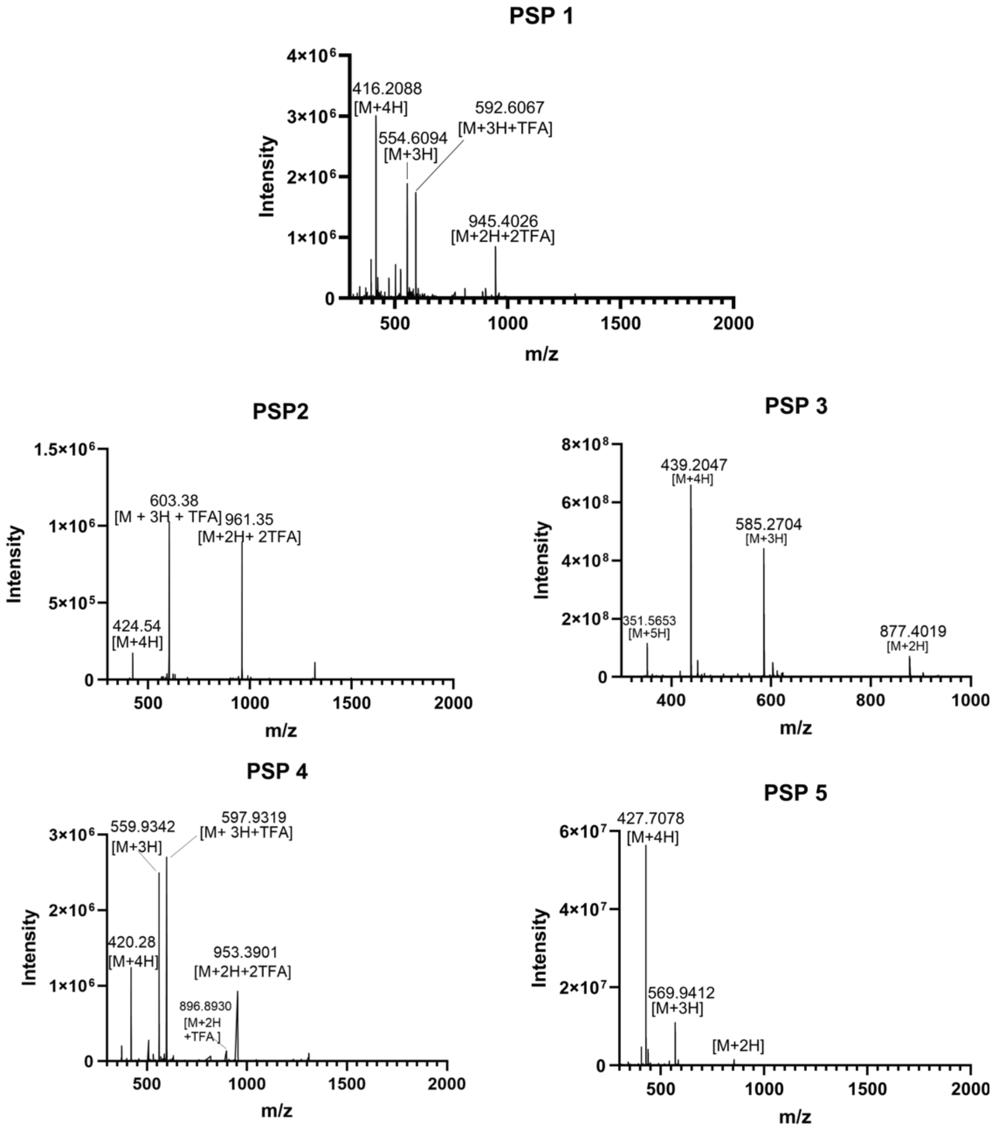

Figure S1. Peptide purity. Electrospray ionization (ESI)- mass spectrometry (MS) of purified peptides utilized in this study. The peptides were prepared at concentrations of 0.05 mg/mL in 50:50 acetonitrile : water with 0.1% formic acid, measured on a Thermo Scientific Orbitrap Exploris™ 240 mass spectrometer. The m/z values obtained showed less than 0.1% deviation from the calculated theoretical mass for the respective peptides.

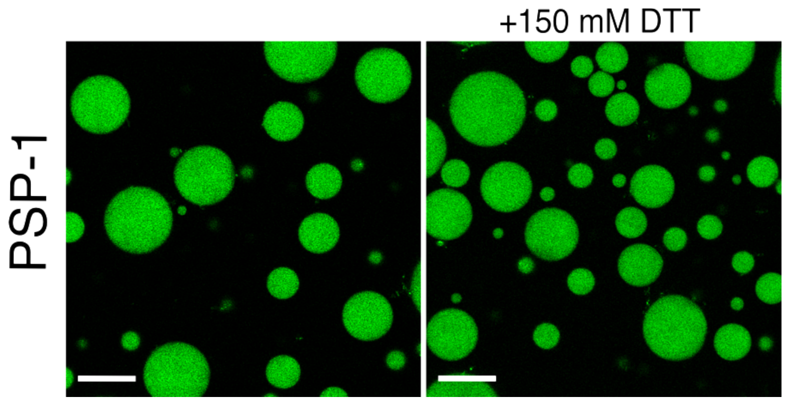

Figure S2. The addition of DTT does not change PSP-1 droplet morphology. PSP-1 self-coacervates (80 mM peptide in 50 mM Tris, 2.5 M NaCl, pH 8.0) with and without DTT. The droplets are formed in the conditions identical to those mentioned in Figure 2.

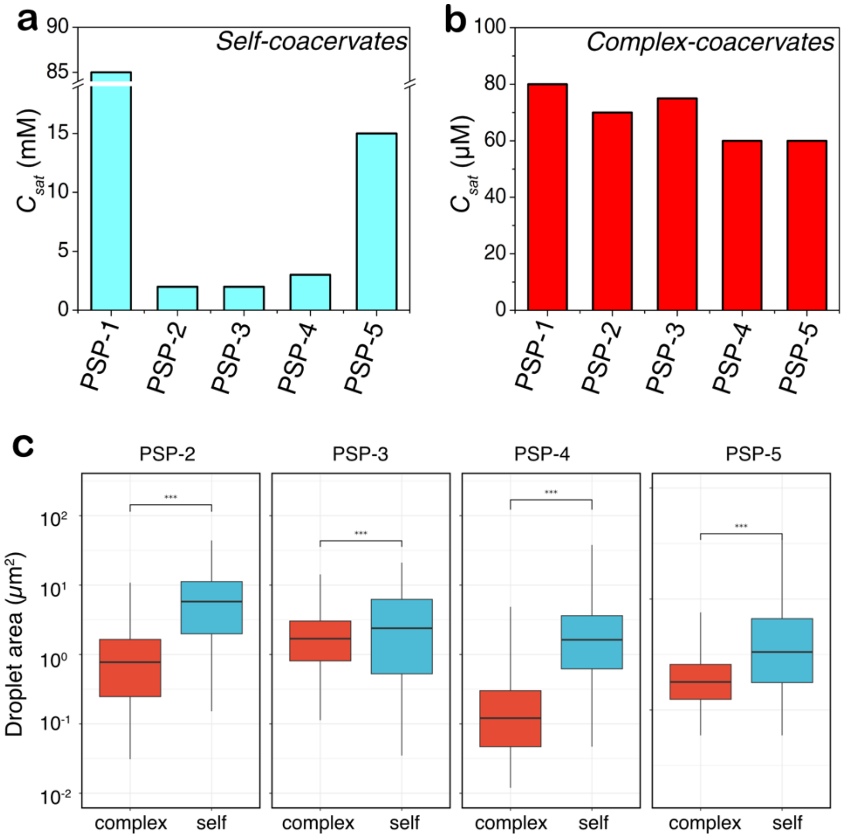

Figure S3: **Self- and complex-coacervates show distinct differences in C_{sat} and their droplet sizes**. C_{sat} values derived from Figures 2 and 7 for self- (a) or complex-coacervation (b). (c) Box and whisker plots of peptide droplet surface area of complex- and self-coacervates obtained from confocal images. (*n*= 5; *** = p> 0.01).

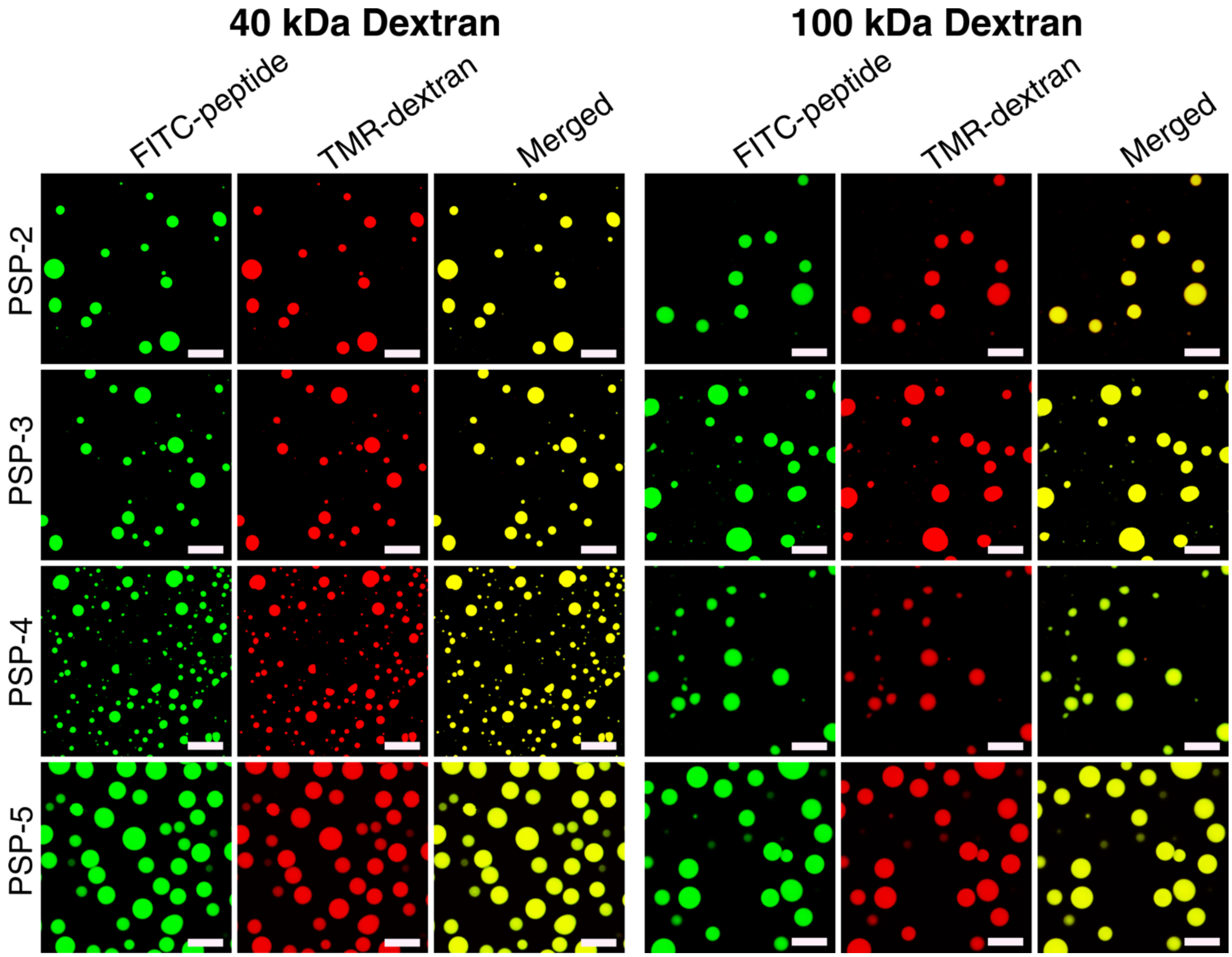

Figure S4. Evaluation of pore size by fluorescent dextran beads. As described in the Methods section, each peptide labeled with FITC at a 1% label ratio (350 µM final concentration) was incubated to form droplets in respective phase separating conditions, i.e., 3.5 mM peptide concentrations (except PSP-5, 18 mM) at pH 8.0 with 50 mm Tris, 2.5 M NaCl (except PSP-5, 3 M). TMR-labeled Dextran beads of two different molecular weights, 40 and 100 kDa were then added to the droplets at a final concentration of 0.5µM (Left and right panels, respectively). The droplets were visualized by confocal microscopy to observe the permeability of Dextran beads to extract information on the porosity of the droplets. The scale bar represents 20 µm.
